# Mechanisms and clinical progress of spinal cord stimulation in refractory chronic pain: an overview

**DOI:** 10.3389/fneur.2025.1687276

**Published:** 2026-01-16

**Authors:** Bo Zhong, Xiaodong Shi, Xunhui Yuan, Yanhong Liu

**Affiliations:** 1 Department of Neurosurgery, Yidu Central Hospital of Weifang, Weifang, China; 2 Department of Pain Management, Yidu Central Hospital of Weifang, Weifang, China

**Keywords:** mechanism, neurotransmitters, pain, SCS, transduction pathways

## Abstract

Chronic pain is a major global health issue, affecting approximately 25% of the population. Managing this condition remains challenging due to the limited efficacy of current therapeutic options. Spinal cord stimulation (SCS), a form of neuromodulation, has been utilized to treat intractable visceral pain. This review summarizes recent advancements in understanding the effects and mechanisms of SCS in refractory chronic pain. Key mechanisms include neurotransmitter modulation, descending inhibition, and cortical changes. SCS operates through various modes, such as high-frequency, burst, closed-loop, dorsal horn inhibition, and descending control. Clinical indications for SCS encompass Failed Back Surgery Syndrome (FBSS), Complex Regional Pain Syndrome (CRPS), Painful Diabetic Peripheral Neuropathy (PDPN), ischemic pain, and cancer pain. This article aims to explore the clinical potential of SCS and the mechanisms underlying its therapeutic efficacy.

## 1 Introduction

Chronic pain is a widespread and debilitating condition, affecting an estimated 25% of the global population, with a particularly severe impact in cases of refractory pain. It is a leading cause of disability and a significant contributor to the global disease burden (1). The incidence of chronic pain varies globally, influenced by factors such as demographics, socioeconomic status, and geographic location (2). In the United States, chronic pain is notably more prevalent, especially among women and certain at-risk populations, posing substantial public health and socioeconomic challenges (3). Research indicates that the direct medical costs and indirect productivity losses associated with chronic pain represent major economic burdens (4). Despite the availability of various treatments, many patients continue to face barriers in accessing effective pain management, exacerbating its economic impact, particularly among middle-aged and elderly individuals (5–7). Consequently, improving the treatment and management of chronic pain is critical.

The treatment of chronic refractory pain remains a significant challenge in the medical field. Traditional drug or surgical interventions often fail to provide relief in cases of chronic or refractory pain. However, recent advancements in the understanding of pain mechanisms have led to the development of new treatment options, offering renewed hope to patients. Spinal cord stimulation (SCS), a neuromodulation technique, has been employed to treat intractable visceral pain, though current evidence does not yet support its routine use as a standard treatment (8). SCS achieves analgesia by delivering electrical stimulation to the dorsal column of the spinal cord, while traditional low-frequency stimulation often results in sensory disturbances. In contrast, newer stimulation patterns such as high-frequency and burst stimulation have demonstrated enhanced analgesic effects without relying on sensory abnormalities (9, 10). High-frequency 10 kHz SCS, a novel technology, selectively activates inhibitory interneurons in the spinal cord’s dorsal horn using low-intensity stimulation, thereby providing pain relief without sensory side effects (11–13) (Clinical applications and manufacturers of different types of SCS are listed in Table 1). This technique has shown significant clinical efficacy in treating chronic back pain and neuropathic pain (14, 15). Burst stimulation is also believed to provide analgesia through distinct mechanisms, though its exact mechanisms remain unclear (16, 17). Recent large-scale studies have confirmed the effectiveness of SCS in alleviating chronic back and leg pain in a large, double-blind, randomized controlled trial (RCT) (18), as well as in treating Painful Diabetic Peripheral Neuropathy (PDPN) through a prospective two-center clinical trial (19) and chronic pain through propensity-matched retrospective comparative effectiveness research (20).

**Table 1:** Clinical applications and manufacturers of different types of spinal cord electrical stimulation.

Stimulation type	Core technical feature	Patient sensation	Main advantages	Representative companies
Traditional SCS	Fixed frequency, continuous stimulation	Distinct paresthesia (tingling)	Mature technology, long history of use	Medtronic, Boston Scientific
High-frequency SCS	Very high frequency (10 kHz)	No paresthesia	Paresthesia-free therapy, effective for back pain, low posture sensitivity	Nevro
Burst SCS	Clustered pulses, mimics brain firing	No or minimal paresthesia	May improve pain affect, paresthesia-free therapy	Abbott
Closed-loop SCS	Real-time monitoring and adjustment of stimulus intensity	Can be configured with or without sensation	Stable efficacy, highly personalized, resistant to interference	Saluda Medical
DTM SCS	Mixed modes, modulates cellular networks	Typically no sensation	Multi-mechanism action, potentially more effective for refractory back pain	Medtronic

In conclusion, electrical stimulation techniques present multiple options for managing chronic refractory pain. With ongoing technological advancements, the effectiveness and applicability of these methods are expected to improve further. This review explores the potential mechanisms by which SCS alleviates chronic refractory pain and examines recent clinical progress (see Table 2).

**Table 2:** Clinical indications of SCS.

Indication	Typical response	Evidence quality	Limitation
Failed Back Surgery Syndrome (FBSS)	50–70% pain reduction, improved function, reduced medication use	High	Not 100% effective, hardware/surgical risks, high cost
Complex Regional Pain Syndrome (CRPS)	Significant relief of burning pain and allodynia, improved blood flow and function	High	Primarily targets pain, underlying condition may progress, complication risks
Painful Diabetic Neuropathy (DPN)	>80% of patients achieve >50% pain relief, improvement in paresthesia	Medium	Higher infection risk in diabetics, requires multidisciplinary management
Postherpetic Neuralgia (PHN)	Variable; can be effective for burning pain and allodynia	Medium	Relatively limited evidence, older patient population with comorbidities, unpredictable efficacy
Refractory angina	Reduced attack frequency and severity, increased exercise tolerance, improved quality of life	High	Does not treat coronary stenosis, requires strict patient selection, theoretical risk of masking myocardial infarction

## 2 Principles and techniques of SCS

### 2.1 Placement and parameter setting of stimulation electrode

**Figure 1:**
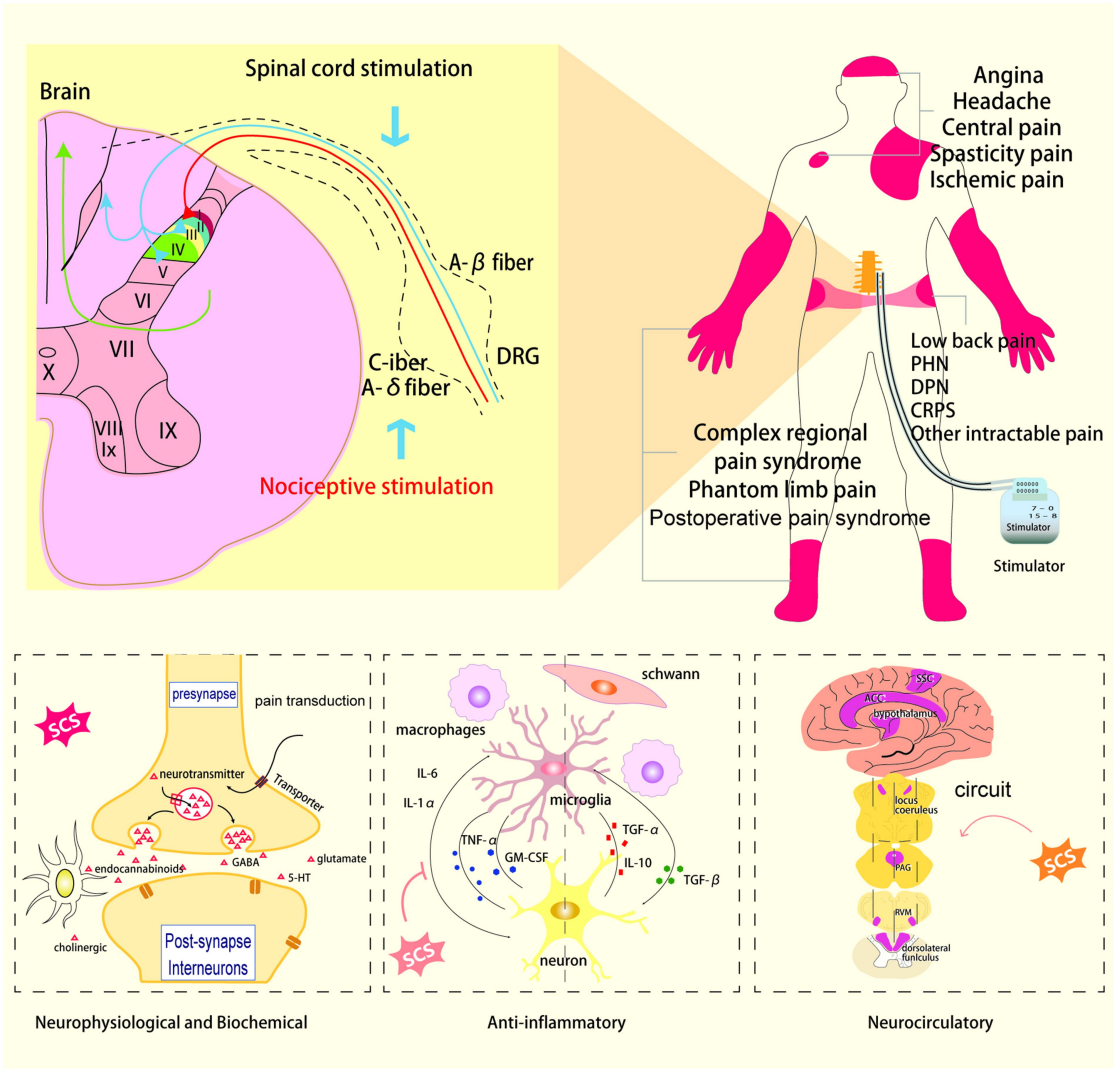
Schematic drawing showing the site of action and clinical indications of spinal cord stimulation.

Spinal cord stimulation is a proven method for managing chronic pain, with its efficacy largely dependent on the precise placement of the stimulating electrodes and the parameters set. The positioning and geometry of the electrodes significantly influence the effectiveness of the stimulation. Electrode placement, geometry, and polarity are critical factors in determining the activation characteristics of cortical neurons during epidural percutaneous electrical stimulation (ECS) (21). Furthermore, a shorter distance between the electrode and the spinal cord results in broader sensory coverage and reduced energy consumption (22). In SCS, electrode placement plays a pivotal role in determining the selectivity and efficiency of stimulation. Positioning the electrodes within the dura mater enhances stimulation efficiency and decreases the power required to stimulate the dorsal column (23). This approach also improves selectivity, enabling the activation of a larger proportion of dorsal column fibers before they diffuse to the dorsal root fibers (23). The selection of electrode configuration and stimulation parameters is essential for optimizing SCS outcomes. Altering the cathode current ratio in bipolar electrode configurations can control the recruitment of dorsal column and dorsal root fibers, thereby modulating the sensory coverage area (24). Moreover, adjustments in electrode configuration can enhance airway patency, a factor independent of voltage increase (25). Clinically, the placement and settings of the electrodes must be tailored to each individual’s physiological state to achieve the most effective therapeutic results. In a prospective, multicenter feasibility study, stimulation settings typically included a frequency of 10 kHz, a pulse width of 30 ms, with amplitudes adjusted to optimize pain relief (26). A multicenter study showed that high-frequency cervical SCS at 10 kHz effectively treats intractable neck and upper limb pain with stable, long-term outcomes (27). Chapman et al. (28) further reported that dorsal root ganglion stimulation (DRG-S) offers an effective treatment for chronic axial low back pain (see Figure 1).

### 2.2 Mode and frequency of SCS

Traditional SCS typically utilizes low-frequency stimulation to induce sensory abnormalities for pain relief. However, advancements in technology have led to the development of high-frequency and non-sensory stimulation patterns, which have demonstrated promising analgesic effects. High-frequency SCS, such as 10 kHz, effectively alleviates pain without causing sensory disturbances (29, 30). The introduction of closed-loop SCS systems has further expanded the possibilities for pain management. These systems maintain spinal cord activation within a defined therapeutic window by monitoring induced compound action potentials (ECAPs), enabling personalized pain management (18). A multicenter, open-label Avalon study confirmed that closed-loop systems provide significant long-term pain relief, with many patients reducing opioid use following treatment (31). Low-frequency and high-frequency stimulation each modulate spinal cord neuronal activity in distinct ways. High-frequency SCS likely involves blocking axonal action potential conduction, altering neuronal excitability, and regulating glial cell function, without relying on the gate control theory. This approach features relatively simple programming, with treatment plans primarily determined by electrode placement and the pain area, and does not require real-time feedback from the patient (32). Low-frequency stimulation inhibits pain transmission by affecting local spinal circuits and dorsal column fibers, while high-frequency stimulation may produce analgesia through different mechanisms (33).

**Figure 2:**
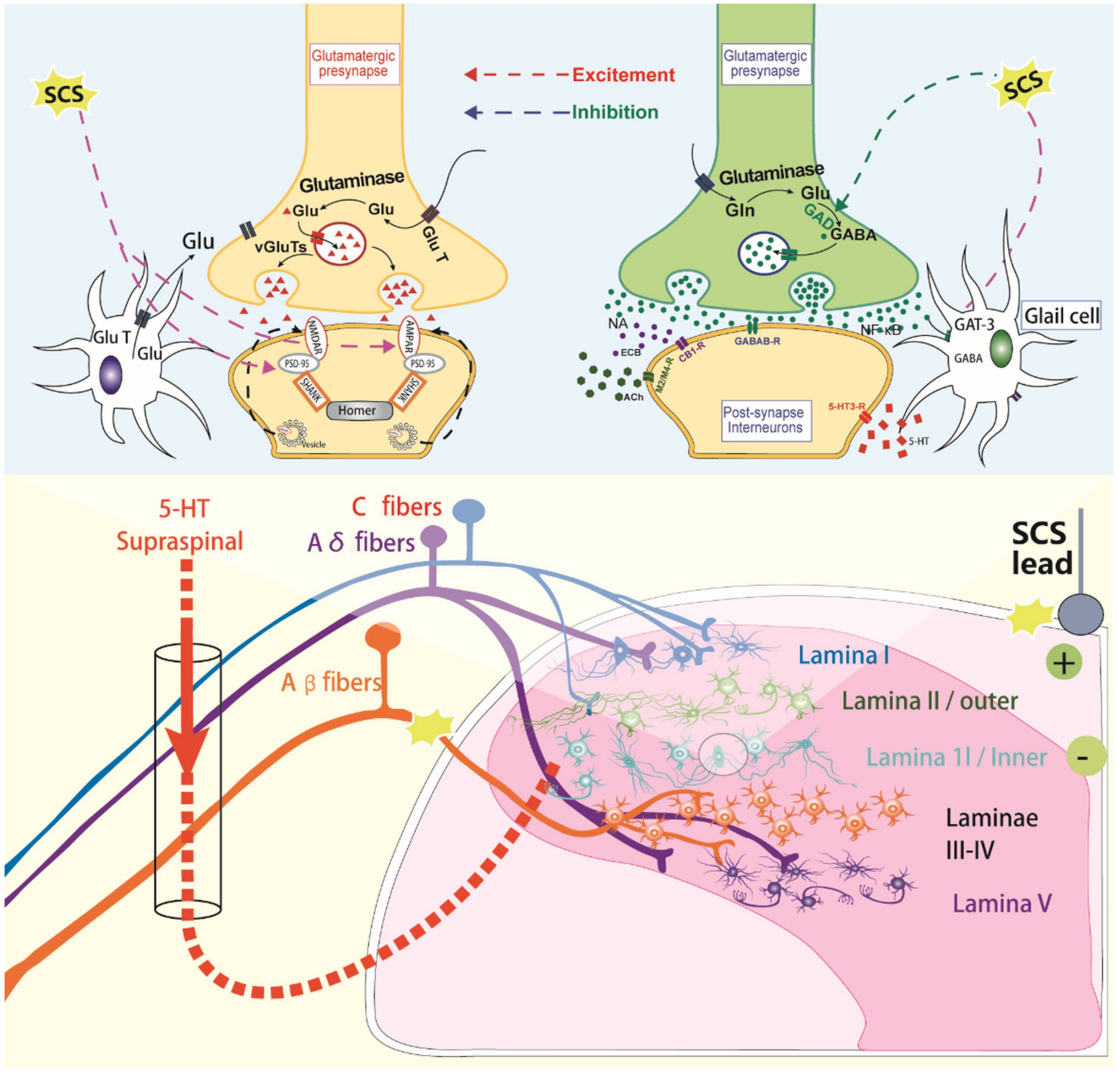
The molecular mechanisms of spinal cord stimulation.

High-frequency stimulation reduces the activation threshold of dorsal column fibers, altering neuronal response patterns and providing a distinct pain relief mechanism compared to traditional low-frequency stimulation (34). Furthermore, research indicates that high-frequency stimulation provides pain relief without discomfort, making it an attractive alternative treatment option (35). These findings offer valuable insights into the neurophysiological mechanisms of SCS and guide the future development of these systems. In conclusion, innovations in SCS modality and frequency present diverse options for chronic pain management. By optimizing stimulation parameters and integrating new technologies, SCS is poised to offer effective pain relief for an increasing number of patients in the future (see Figure 2).

## 3 Mechanism of SCS in refractory chronic pain

### 3.1 Effects of SCS on neurotransmitters

Spinal cord stimulation, as a neuromodulation technique, has been utilized in the treatment of various chronic pain conditions and neurological dysfunctions. Research indicates that SCS can modulate the release of neurotransmitters and neuronal excitability through multiple mechanisms, thereby improving patient symptoms. Firstly, SCS can influence neurotransmitter release by modulating neural circuits within the spinal cord. For instance, studies suggest that SCS may alleviate chronic neuropathic pain by promoting the release of the inhibitory neurotransmitter GABA (9). Additionally, SCS regulates the excitability of motor neurons by modulating the release of norepinephrine (NA), which in turn affects the occurrence of muscle spasms, as observed in rat models of chronic spinal cord injury (SCI) (36). Secondly, the effect of SCS on neurotransmitters is also evident in its ability to regulate neuronal excitability. SCS influences neuronal excitability by modulating the activity of calcium ion channels, altering the pattern of neurotransmitter release (37). This modulation is not confined to the spinal cord but extends to neural activity in the cerebral cortex (38). Xiang et al. (39) further reported that signaling through spinal A1R and A3R receptors provides sustained suppression and contributes to SCS-induced inhibition of spinal pain transmission following nerve damage. Blocking adenosine deaminase may serve as a complementary treatment to enhance the pain relief effects of SCS. Moreover, SCS can facilitate the recovery of neurological functions by influencing the neural network of the spinal cord. For example, SCS can activate reflex circuits within the spinal cord by stimulating sensory nerve roots, thereby improving motor function (40). This mechanism is likely linked to SCS’s regulation of neurotransmitter release. Lastly, SCS’s effects may also be related to its influence on neurotransmitter-related signaling pathways. Research suggests that SCS can promote neuroregeneration and functional recovery by modulating protein expression in specific signaling pathways (41). This provides new insights into using SCS for the rehabilitation of neurological injuries. In conclusion, SCS plays a critical role in treating chronic pain and neurological dysfunction by modulating neurotransmitter release and neuronal excitability through diverse mechanisms. These findings offer a theoretical foundation for the further optimization of SCS treatment strategies.

### 3.2 SCS regulates neurotransmitter receptor expression

Spinal cord stimulation is an effective treatment for chronic pain, achieved through electrical stimulation of the dorsal column of the spinal cord to alleviate discomfort. Research indicates that SCS exerts its analgesic effects by regulating the expression of neurotransmitter receptors. For instance, SCS can modulate the activity of N-methyl-D-aspartate (NMDA) receptors in the spinal cord, thereby reducing neuropathic pain (42). Additionally, SCS can induce a long-term reversal of pain hypersensitivity by influencing the endocannabinoid system, particularly the CB1 receptor (43). In chronic pain management, SCS not only affects neurotransmitter receptors in the spinal cord but also restores the balance between pain input and inhibition by modulating pain processing pathways in the brain. Burst SCS, for example, significantly alters EEG activity in brain regions associated with pain processing, leading to a reduction in chronic neuropathic pain (44). Furthermore, SCS can alleviate pain by modulating neuroinflammatory processes in the spinal cord. Studies have shown that SCS reduces neuropathic pain by regulating MAP kinase and NF-κB signaling pathways to mitigate neuroinflammation (45). Fei et al. (46) demonstrated that spinal CB1 receptor activation plays a role in synaptic depression of high-threshold afferent inputs in substantia gelatinosa (SG) neurons following Aβ-ES and may be involved in the SCS-induced suppression of spinal nociceptive transmission after nerve injury. This regulatory effect is likely linked to SCS’s impact on neurotransmitter receptor expression. The therapeutic effect of SCS is also influenced by psychological factors. Conditions such as depression and anxiety can affect the outcomes of SCS treatment, highlighting the need for psychological evaluation prior to therapy (47). Bendinger et al. (48) reported that psychological factors, including sleep disturbances, serve as independent risk factors for unsuccessful SCS outcomes. A retrospective study of 60 consecutive patients found that factors such as the Hospital Anxiety and Depression Scale (HADS-D), Beck Depression Inventory-II (BDI-II), and Pain Disability Index (PDI) scores were inversely correlated with the effectiveness of SCS therapy (49).

In summary, SCS treats chronic pain through a range of mechanisms, including the modulation of neurotransmitter receptor expression and the regulation of neuroinflammatory processes. This multi-faceted approach makes SCS a highly effective tool in chronic pain management.

### 3.3 Segmental and supraspinal modulation of pain by SCS

Spinal cord stimulation is a highly effective treatment for chronic pain, particularly when traditional therapies fail. By applying electrical current to the dorsal columns of the spinal cord, SCS alleviates pain through the modulation of pain conduction pathways. Studies have demonstrated that SCS reduces pain through several mechanisms, including the modulation of pain processing pathways in the central nervous system (50). SCS influences neuronal circuits in the dorsal horn of the spinal cord, reducing the transmission of pain signals. By inhibiting the excitability of neurons in this region, SCS diminishes the perception of pain (51). Additionally, SCS enhances endogenous pain inhibition by modulating the descending pain inhibitory pathway, further alleviating pain (52). Barchini et al. (53) found that SCS activates both supraspinal and segmental mechanisms. In models with dorsal column lesions, rostral and caudal stimulations engage distinct synaptic pathways and neurotransmitter systems. Heijmans and Joosten (9) also reported that tonic SCS primarily acts through a segmental spinal mechanism, stimulating GABA release from inhibitory interneurons in the spinal dorsal horn. Tonic SCS simultaneously triggers neuropathic pain modulation *via* a feedback loop between the supraspinal and spinal regions, involving descending serotonergic fibers. SCS has been shown to reduce pain by decreasing the overexcitation of spinal neurons and modulating the transmission of pain signals (54). Moreover, SCS regulates both ascending and descending pain pathways to achieve analgesia (55). Although the precise mechanism by which SCS inhibits pain transduction remains unclear, research suggests that it modulates neural circuits in both the spinal cord and brain (56). SCS also alleviates neuropathic pain by inhibiting microglial activation in the dorsal horn of the spinal cord (57). Clinically, the effects of SCS extend beyond the spinal level to include supraspinal mechanisms. For example, SCS regulates the balance between pain input and inhibition in the brain, restoring normal pain processing in patients with chronic neuropathic pain (44). SCS can also inhibit pain by modulating neuronal activity in the spinal cord. Additionally, SCS reduces pain transmission by regulating GABA receptor signaling pathways in the dorsal horn of the spinal cord, thereby alleviating pain. This effect not only suppresses pain but also reduces arrhythmias associated with chronic pain (58). In summary, SCS activates pain inhibition networks in both the spinal cord and brain, modulating neuronal activity and reducing sympathetic nerve excitation through GABA signaling pathways. The combined action of these mechanisms makes SCS an effective treatment for chronic neuropathic pain.

### 3.4 SCS regulates brain function

Spinal cord stimulation, as a neuromodulation technique, has been widely used in chronic pain management. However, its precise mechanisms of action on brain function remain poorly understood. Research suggests that SCS may alleviate pain by modulating the brain’s pain processing pathways. The mechanisms could involve local spinal cord changes or broader neural networks, including subcortical and cortical brain regions (59). Different SCS modes, such as burst and high-frequency, trigger varying temporal activity patterns that ultimately produce specific effects on pain inhibition. High-density electroencephalogram (EEG) studies have shown that SCS significantly reduces *θ* and low *α* band (6–10 Hz) activity, which is associated with pain, supporting the thalamic-cortex rhythm disorder hypothesis (59). These findings suggest that SCS’s analgesic effect may be linked to its ability to enhance endogenous pain inhibition. In one study, SCS significantly activated endogenous pain modulation systems, particularly improving dynamic pain measures such as Conditioned Pain Modulation (CPM) efficiency and time-averaged pain responses (50). The analgesic effect of SCS extends beyond single sensory parameter changes, exhibiting a general anti-hypersensitivity effect on abnormal pain. This suggests that SCS may regulate central nervous system circuits to exert its therapeutic impact (52). In patients with chronic neuropathic pain, SCS also demonstrates a restorative effect on the imbalance between pain input and inhibition in the brain. Studies indicate that SCS achieves this by altering brain electrical activity and connectivity, thereby partially restoring normal brain function (44). Consequently, SCS affects brain function through multiple mechanisms, playing a pivotal role in chronic pain management. These mechanisms include modulating pain processing pathways, activating the endogenous pain suppression system, and influencing brain activity and connectivity (44, 50, 52, 59). These findings provide a crucial theoretical foundation for further research into the application of SCS in pain management. Bu (60) also reported that SCS induces alterations in dALFF in patients with postherpetic neuralgia (PHN), suggesting its potential to modify brain function and alleviate pain, sleep disturbances, and mood symptoms. These findings offer new insights into the mechanisms underlying SCS effectiveness in treating PHN. Peter (61) was the first to describe altered cross-network functional connectivity in emotion and reward circuits of the brain in chronic pain patients with fully implanted SCS systems, as observed using a 3 T MRI scanner. Furthermore, associative plasticity in the spinal cord enhances sensorimotor connections, contributing to some recovery of reflex control and forelimb function following moderate SCI (62). Xiaochong et al. (63) also demonstrated that SCS causes shifts in regional homogeneity and degree centrality in PHN patients, suggesting its ability to influence brain function to reduce pain, improve sleep, and manage emotional disorders. Thus, while the specific regulatory mechanisms remain unclear, SCS likely improves chronic pain by modulating brain function.

### 3.5 SCS modulates neuroplasticity in the brain

Spinal cord stimulation is a technique used to treat chronic neuropathic pain by delivering electrical currents to the dorsal columns of the spinal cord, thereby alleviating pain. Recent studies have demonstrated that SCS not only directly modulates neural activity in the spinal cord but also influences neural plasticity in the brain (44). The mechanisms underlying SCS involve multiple neural pathways, including the reorganization of pain conduction and inhibitory descending pathways (44). In the context of chronic neuropathic pain, SCS alleviates pain by altering brain function and metabolic activity. Research indicates that SCS can partially restore the functional and metabolic activities of brain structures involved in pain perception, emotional responses, and behavioral reactions (64). Furthermore, SCS can impact pain and motor dysfunction by regulating neural connectivity in the brain (65). Another key role of SCS is its ability to improve pain symptoms by modulating neural plasticity both in the spinal cord and brain. Studies have shown that SCS promotes nerve regeneration and functional recovery by enhancing the neural plasticity of the spinal cord (66). Additionally, SCS influences pain transmission and perception by regulating brain plasticity (56). In conclusion, SCS not only alleviates pain through direct effects on the spinal cord but also exerts a broader impact by regulating brain neural plasticity. This multi-level mechanism positions SCS as an effective approach for treating chronic neuropathic pain (44, 56, 64–66).

## 4 Main clinical application scenarios

### 4.1 Neuropathic pain

In clinical practice, SCS has become widely used to treat various chronic pain syndromes, particularly when traditional treatments fail. SCS effectively reduces neuropathic pain and enhances patients’ quality of life (10). SCS is also an effective treatment for post-herpetic neuralgia (PHN), one of the most common complications following herpes zoster infection, often causing persistent pain. A cohort study analyzing data from 2005 to 2014 found that the overall incidence rate of PHN in 2,778,476 adults was 4.92/1000 person-years (PY) [95% confidence interval (CI): 4.86–4.98], with an increased incidence associated with age (67). When conservative treatments fail, SCS remains a promising interventional option (68). In treating PHN, SCS alleviates pain through electrical stimulation of the spinal cord. However, compared to other neuropathic pain conditions, traditional SCS faces significant challenges in providing long-term, stable pain relief for PHN patients. New SCS methods, such as high-frequency stimulation, burst stimulation, and DRG-S, offer promising alternatives by avoiding the painful sensory abnormalities often experienced by PHN patients (69). Further research has validated the use of SCS in PHN. For instance, a study exploring the effectiveness of SCS and pulsed radiofrequency (PRF) in treating herpes zoster-related pain in elderly patients found that both treatments could effectively alleviate PHN pain (70). A prospective RCT (ChiCTR2100050647) involving 44 PHN patients showed that SCS had superior efficacy and safety compared to PRF (71). Another trial with 70 elderly PHN patients demonstrated that while both PRF and SCS are safe and effective neuromodulation treatments, SCS provided longer-lasting and more significant analgesic effects than PRF (72). Similar findings were reported in a randomized, double-blinded controlled trial (73) involving 96 patients with acute/subacute PHN and another trial (70) with 63 patients aged over 50 years. Both studies indicated that SCS and PRF are safe and effective for treating zoster-related pain, with SCS offering greater pain relief and improved quality of life compared to PRF. These findings highlight the clinical effectiveness of SCS in treating PHN and suggest its potential as an adjunct or alternative to pharmacological treatments in certain cases. SCS shows strong prospects in managing PHN, particularly when traditional treatments are ineffective. However, limitations of these studies include small sample sizes and single-center designs, indicating a need for further research to validate and optimize the application of new SCS technologies in PHN treatment.

SCS is gaining recognition for its application in treating Diabetic Peripheral Neuropathy (DPN), particularly the 10 kHz high-frequency SCS, which has received CE approval in Europe for the management of painful diabetic neuropathy. Although it has not yet been directly labeled in the United States, its widespread clinical use suggests that formal approval is forthcoming. SCS can effectively alleviate pain and enhance the quality of life for patients with DPN. In a prospective, open-label study, high-frequency SCS at 10 kHz showed significant effectiveness in relieving pain associated with PDPN, with positive impacts on objective, quantitative measures of nerve function (74). Another study explored the effects of high-frequency SCS on peripheral nerve function in patients with painful diabetic neuropathy. After 12 months of follow-up, patients experienced notable improvements in lower limb pain, weakness, and sensory symptoms, as well as an increase in neuropathy symptom scores. A prospective cohort study conducted in an outpatient clinical setting also provided preliminary data supporting the positive effects of SCS on peripheral neuropathy (75). The use of SCS in treating painful diabetic polyneuropathy also shows promising results. In a pilot study, SCS was found to be a feasible and effective treatment, providing sustained pain relief and improving quality of life over a 12 month follow-up (76). These findings highlight the potential clinical value of SCS in treating DPN, highlighting the need for further research and exploration to optimize its application.

Complex Regional Pain Syndrome (CRPS) is a chronic pain condition that typically affects the limbs and is often challenging to manage effectively with conventional treatments. In such cases, SCS has proven to be an effective treatment option. By implanting a nerve stimulator, SCS alleviates pain and enhances the patient’s quality of life (77). The application of SCS in CRPS patients can lead to significant reductions in pain scores and improvements in patient satisfaction. For instance, a retrospective multicenter study involving 101 patients with CRPS Type I demonstrated a significant reduction in pain scores, with the use of multi-electrode leads proving more effective than single-electrode leads (78). Additionally, a retrospective cohort study of 51 CRPS patients implanted with an SCS system highlighted the long-term effectiveness of SCS in managing CRPS pain (79). High-frequency SCS (HF10-SCS), an emerging treatment, has also yielded promising results in CRPS patients. In a small case series of 13 CRPS patients, 12 received permanent implantation, and 67% of these patients reported a reduction in pain intensity by more than 50% during follow-up (80). A recent prospective multicenter feasibility study involving 16 patients with intractable chronic severe lower limb pain associated with CRPS further demonstrated the functionality, effectiveness, and safety of intraspinal stimulation of dorsal nerve roots in managing this condition. However, most of these studies were conducted at single centers with relatively small sample sizes, and the stimulation methods for SCS were generally simple. Future research should explore the effects of various SCS techniques in CRPS patients to further optimize treatment regimens.

### 4.2 Postoperative pain syndrome

Spinal cord stimulation is an effective technique for managing chronic neuropathic pain, particularly in the treatment of postoperative pain syndrome, also known as Failed Back Surgery Syndrome (FBSS). FBSS is a common and persistent pain condition that typically develops after spinal surgery. SCS alleviates pain by delivering electrical currents to the dorsal columns of the spinal cord and has gained increasing attention in clinical practice in recent years (81). SCS significantly improves pain intensity and quality of life in FBSS patients. SCS not only effectively reduces pain but also decreases anxiety and depressive symptoms, thus enhancing overall well-being. Additionally, SCS has demonstrated potential in reducing opioid use, which is particularly beneficial for long-term analgesic users (82). An integrative review of both quantitative and qualitative studies confirmed that SCS has positive effects across various aspects of life for FBSS patients (83). In an uncontrolled, open-label, prospective study involving 60 FBSS patients, the use of 10 kHz SCS was shown to be safe and effective, significantly improving the quality of life, particularly in patients with radicular symptoms (84). Another multicenter RCT (NCT01697358) enrolled 218 patients and found that integrating multicolumn SCS into osteopathic manipulative medicine (OMM) improved pain relief, quality of life, and functional outcomes in FBSS patients with predominant lower back pain, a traditionally challenging patient group. These improvements were sustained for 12 to 24 months, although SCS-related adverse events may be higher (85). Given the limited current research, further advanced evidence-based studies are needed to confirm the long-term safety and efficacy of SCS in the management of FBSS.

### 4.3 Phantom limb

Spinal cord stimulation is a neuromodulation technique used to manage chronic pain. In recent years, it has gained increasing attention in the treatment of phantom limb pain (PLP) and residual limb pain (RLP). PLP is a common complication following amputation, affecting the majority of amputees, while RLP pertains to discomfort at the amputation site. SCS can alleviate these pain symptoms by modulating neural activity in the spinal cord. Research into the impact of SCS on PLP has demonstrated that SCS effectively reduces pain scores and improves quality of life (86). In a study using a non-invasive percutaneous SCS method, participants showed a significant reduction in McGill pain questionnaire scores after just 5 days of treatment, indicating a substantial short-term alleviation of PLP. Additionally, another study supports the role of SCS in managing PLP, suggesting that SCS influences pain perception by modulating spinal cord excitability, thus reducing the severity of the pain (87). While the precise mechanisms of SCS remain unclear, it is widely believed to alleviate pain by affecting central nervous system plasticity. However, despite promising results in some patients, the application of SCS in phantom limb and RLP requires further investigation. Most existing studies are preliminary and involve small sample sizes, highlighting the need for large-scale RCTs to confirm its long-term efficacy and safety (88). In conclusion, as a non-pharmacological treatment, SCS presents a novel therapeutic option for managing phantom limb and RLP. Nevertheless, its clinical application requires careful evaluation and further scientific validation.

### 4.4 Ischemic pain

In the treatment of ischemic pain, the mechanism of action of SCS is likely related to its regulation of neural transmission. By modulating the transmission of neural signals between the spinal cord and the brain, SCS helps balance pain input and inhibitory pathways, thereby reducing the perception of pain (44). Additionally, SCS may promote the healing of ischemic wounds by improving local blood flow and tissue oxygenation, providing theoretical support for its use in ischemic pain management (89). Despite its broad potential in treating ischemic pain, the specific mechanisms of action still require further research. A deeper understanding of how SCS affects the spinal cord and brain will help optimize therapeutic strategies and expand its application in pain management (56). Future studies should explore the efficacy of SCS across different types of ischemic pain, as well as its synergistic effects with other treatments.

In recent years, SCS has also garnered increasing attention in the management of diabetic foot pain, a common complication in diabetic patients that significantly impacts quality of life. Traditional drug treatments often have limited effectiveness and may cause adverse reactions, highlighting the need for new therapeutic approaches. SCS has shown significant effectiveness in alleviating pain from PDN. A systematic review and meta-analysis revealed that, compared to optimal drug therapy, SCS significantly reduces pain intensity in PDN patients and improves health-related quality of life (90). Another study suggests that SCS can effectively relieve mechanical and thermal hyperalgesia in DPN patients, potentially by inhibiting neuroinflammation in the dorsal horn of the spinal cord (91). Moreover, SCS has demonstrated potential benefits in treating diabetic foot ulcers. A retrospective cohort study comparing short-term SCS treatment with debridement showed that SCS provided significant early advantages in ulcer healing, pain relief, improved circulation, and reduced amputation rates (92). However, these benefits did not persist beyond 6 weeks, indicating the need for further research to optimize treatment regimens. Mechanistic studies also support the application of SCS in diabetic foot pain management. Research suggests that SCS alleviates pain by modulating neural pathways in the spinal cord and brain (44). Furthermore, different SCS stimulation modes, such as Differential Targeting Multiplexing (DTM), may offer varying effects in relieving spontaneous neuropathic pain, presenting opportunities for personalized treatment (93).

Spinal cord stimulation has demonstrated efficacy in treating refractory angina, a condition characterized by persistent angina despite optimal anti-anginal therapy and an inability to be relieved through revascularization surgery. In a multicenter randomized single-blind study with a small sample size, researchers compared sensory SCS, subconscious SCS, and sham SCS in patients with refractory angina. The results indicated that sensory SCS significantly reduced the frequency of angina attacks, nitroglycerin usage, and improved quality of life, while subconscious SCS had no notable effect. This suggests that sensory SCS offers a clear advantage in improving the clinical status of patients with refractory angina (94). Furthermore, the application of SCS in angina patients extends beyond pain control; it may also have positive effects on cardiac function. SCS can enhance heart function, inhibit arrhythmias, and regulate the autonomic nervous system, further supporting its use in treating angina (95). Although SCS has shown promising clinical and potential cardioprotective effects for refractory angina, further research is needed to optimize stimulation parameters and evaluate its long-term efficacy.

### 4.5 Other intractable pain

Cancer remains a leading cause of death globally, with pain often being the first symptom of malignant tumors. An increasing number of individuals suffer from cancer-related pain, many of whom fail to achieve adequate relief. Given SCS’s established effectiveness in treating neuropathic pain, it should be considered earlier as an adjunctive therapy for cancer-related pain. Moreover, as cancer treatment advances and survival rates improve, SCS may help reduce the risk of chronic neuropathic pain in cancer survivors (96). In cancer pain management, SCS offers a non-pharmacological alternative. SCS can effectively reduce chronic pain from trauma or neuropathy, and in some cases, it can stabilize or decrease opioid use. The advent of high-frequency SCS modes, such as 10 kHz SCS, has the advantage of being independent of sensory abnormalities. Many patients on high-dose opioids can reduce their intake after starting SCS therapy, lowering their risk and improving the effectiveness of pain relief (97). In addition to pain relief, the application of SCS in cancer pain management may extend to aiding wound healing. Studies suggest that SCS could enhance local blood flow and tissue oxygenation, potentially promoting the healing of ischemic wounds. While this application requires further investigation, case reports have indicated that SCS may not only treat refractory pain but also support the healing of ischemic wounds (89).

Spinal cord stimulation is an effective method for treating chronic central pain. While its efficacy in managing peripheral neuropathic pain is well-established, its application in central pain, particularly post-stroke central pain (CPSP), remains controversial (98). SCS may alleviate central pain by modulating the pain regulatory centers in the spinal cord and brainstem, involving both segmental and supraspinal mechanisms (99). In treating central pain following stroke, the effectiveness of SCS may be influenced by factors such as the patient’s age and the location of the stroke. Younger patients and those whose strokes do not involve the thalamus are more likely to benefit from SCS (98). Additionally, SCS has shown promise in treating central neuropathic pain after SCI. SCS may exert its analgesic effects by modulating inflammatory responses in the spinal cord (100). However, the application of SCS for central pain requires further investigation to better understand its mechanisms and optimize treatment protocols. Despite positive outcomes in some cases, a large retrospective multicenter study cautioned that the use of SCS in central pain should be approached carefully, particularly when dealing with complex central nervous system mechanisms (101). Future research should focus on exploring the mechanisms of SCS in central pain to enhance both its clinical effectiveness and safety.

## 5 Future prospect

Spinal cord stimulation, an effective method for treating chronic intractable pain, has seen significant technological advancements in recent years. However, its’ neurophysiological and biochemical mechanisms are not yet fully understood. Animal studies have provided some insights into the spinal cord and supraspinal mechanisms that may underlie the analgesic effects of SCS (102). Additionally, the mechanisms of SCS extend beyond traditional gating theories; research suggests that the impact of SCS on spinal sensory projection neurons may involve more complex neural circuits (103). In patients with SCI, combining SCS with active training has been shown to promote the recovery of lower limb motor function. Specific SCS parameters can modulate the excitability of spinal circuits, allowing sensory information and residual descending inputs to guide appropriate movement patterns (104). Moreover, SCS may play a role in enhancing neural plasticity, further improving motor function and providing promising directions for future research. An important aspect of SCS is its role in neuromodulation. Studies have shown that SCS can suppress neuro-mechanical hypersensitivity through optimized charge transfer, suggesting that adjustments to electrical stimulation parameters may significantly influence individualized treatment outcomes (105). These findings not only lay a theoretical foundation for the clinical application of SCS but also highlight future research opportunities, particularly in optimizing electrical stimulation parameters to enhance therapeutic effects.

Spinal cord stimulation has made notable progress in recent years in terms of both technological advancements and parameter optimization. The efficacy of SCS is closely linked to stimulation parameters, such as pulse width, frequency, and intensity. Optimizing these parameters can significantly enhance treatment outcomes, with adjustments in pulse width and stimulation amplitude promoting central activation and improving motor function (106). Further breakthroughs in basic research are anticipated. However, in China, the high cost of SCS surgery, which exceeds medical insurance coverage, remains a significant barrier, limiting its clinical application. Inclusion of SCS in medical insurance policies would make this treatment more accessible to a broader population.

The development of closed-loop stimulation systems has enabled real-time monitoring and adjustment of stimulation parameters, enhancing treatment precision and personalization (16). Additionally, the application of computer models has improved our understanding of the electrical properties of intraspinal structures, facilitating the optimization of electrode geometry and configuration (107). However, there remains a lack of research focused on optimizing therapeutic parameters, and evidence-based studies on the treatment parameters for different pain phenotypes are limited. Future clinical studies are needed to address these gaps and refine SCS applications.

Although the clinical application of SCS has demonstrated significant efficacy in some cases, there remains a lack of large-scale, rigorous clinical studies to comprehensively evaluate its long-term effects and safety. Regarding SCS research design, professional organizations such as the International Society for Neuromodulation have made several recommendations to enhance the effectiveness and reliability of RCTs. These include incorporating mechanistic endpoints when possible, avoiding non-inferiority designs without internal sensitivity validation, ensuring double-blind conditions whenever feasible, and utilizing placebo or sham surgery controls, among others (108). Comparative studies of different SCS technologies and devices are also relatively limited. One study compared the effects of SCS devices from different manufacturers on pain relief, sleep, and functional outcomes. The results revealed no significant differences in pain relief or sleep improvement, though differences in functional improvement were observed (109). This suggests that different devices may have varying effects on certain outcomes, but the small sample size warrants caution in drawing firm conclusions. Furthermore, a multicenter international study assessed the impact of multi-column electrode programming on back pain in patients with FBSS. The results indicated that the multi-column configuration was more effective than the single-column configuration in providing pain relief across all anatomical regions after 6 months of follow-up (110). This suggests that multi-column SCS may offer superior pain coverage and relief in certain cases. Despite its promise in chronic pain management, further large-scale and well-designed clinical studies are essential to confirm the long-term efficacy and safety of SCS. These studies will not only optimize the clinical application of SCS but also provide patients with a more reliable and evidence-based treatment option.
